# Shikonin induces mitochondria-mediated apoptosis and enhances chemotherapeutic sensitivity of gastric cancer through reactive oxygen species

**DOI:** 10.1038/srep38267

**Published:** 2016-12-01

**Authors:** Wenquan Liang, Aizhen Cai, Guozhu Chen, Hongqing Xi, Xiaosong Wu, Jianxin Cui, Kecheng Zhang, Xudong Zhao, Jiyun Yu, Bo Wei, Lin Chen

**Affiliations:** 1 Department of General Surgery, Chinese People’s Liberation Army General Hospital, Beijing 100853, P. R. China; 2 Department of Frontier for Biological Treatment, Beijing Institute of Basic Medical Science, Beijing, 100850, China

## Abstract

The prognosis of gastric cancer remains poor due to clinical drug resistance. Novel drugs are urgently needed. Shikonin (SHK), a natural naphthoquinone, has been reported to trigger cell death and overcome drug resistance in anti-tumour therapy. In this study, we investigated the effectiveness and molecular mechanisms of SHK in treatment with gastric cancer. *In vitro*, SHK suppresses proliferation and triggers cell death of gastric cancer cells but leads minor damage to gastric epithelial cells. SHK induces the generation of intracellular reactive oxygen species (ROS), depolarizes the mitochondrial membrane potential (MMP) and ultimately triggers mitochondria-mediated apoptosis. We confirmed that SHK induces apoptosis of gastric cancer cells not only in a caspase-dependent manner which releases Cytochrome C and triggers the caspase cascade, but also in a caspase-independent manner which mediates the nuclear translocation of apoptosis-inducing factor and Endonuclease G. Furthermore, we demonstrated that SHK enhanced the chemotherapeutic sensitivity of 5-fluorouracil and oxaliplatin *in vitro* and *in vivo*. Taken together, our data show that SHK may be a novel therapeutic agent in the clinical treatment of gastric cancer.

Gastric cancer is a malignant cancer developing from epithelial cells of the lining of the stomach. Over past 50 years, the incidence and mortality of gastric cancer were declining due to prevalence of *H. pylori* infections and a better availability of fresh foods^1^, however rates still remain high in low- and middle-income countries^2^. It ranks fifth in tumour incidence rate and third in cancer mortality worldwide^2^,^3^. Although surgery is the main curative therapy for stomach cancer, chemotherapy still has an important role, as many patients have regional or distant spread at the time of diagnosis^4^,^5^,^6^. Fluorouracil or its analogues, platinum or its derivatives, and paclitaxel are regarded as first-line therapies of gastric cancer^7^. Multi-drug resistance (MDR) is one of the main causes of chemotherapeutic failure^8^. Hence, searching for effective chemo-sensitive drugs and overcoming MDR is a looming problem.

Shikonin (SHK), a naphthoquinone derived from the roots of the herbal plant *Lithospermum erythrorhizon*, has been used for thousands of years in ancient China given its anti-inflammatory and anti-microbial activities in treatment of burns, skin diseases and sore throats^9^,^10^. Recently, it has been demonstrated that SHK has significant anti-tumour activities, such as inducing apoptosis in hepatocellular carcinoma, inhibiting melanoma proliferation and killing leukaemia cells^11^,^12^,^13^. SHK also has a cytotoxic effect on MDR cell lines and enhances chemotherapeutic sensitivity. It enhances cisplatin-induced colon cancer cell apoptosis, synergistically kills glioblastoma cells in combination with erlotinib and induces cell cycle arrest in triple negative breast cells^14^,^15^,^16^,^17^. Furthermore, SHK has anti-tumour effects by inducing receptor-interacting protein 1 (RIPK1)-dependent necroptosis^18^,^19^. The anti-tumour abilities of SHK have gained increasing interest among gastric cancer research. For instance, SHK induces apoptosis in the human gastric cancer cells HGC-27 through mitochondria-mediated pathway^20^ and causes cell cycle arrest in human gastric cancer (AGS) by early growth response 1 (Egr1)-mediated p21 gene expression^21^. However, studies about the effects of SHK on gastric cancer are insufficient and its precise anti-carcinogenic mechanisms against gastric cancer warrant further studies before it can be applied into the clinical setting.

Apoptosis, a highly regulated and controlled process, controls embryonic development by inducing the separation of fingers and toes, and removing infected cells to maintain internal environment stability^22^,^23^. Excessive apoptosis causes autoimmune diseases and insufficient apoptosis results in uncontrolled cell proliferation in tumourigenesis^24^,^25^. Apoptosis can be initiated through two pathways: the extrinsic cell death pathway and the intrinsic cell death pathway^26^,^27^. Both pathways induce cell death by activating caspase cascade^28^. Many chemotherapy drugs achieve anti-tumour effects by inducing apoptosis but drug resistance often occurs by caspase escape^29^. Recently, researchers discovered that cells can die and display apoptosis morphology without caspase activation via the translocation of apoptosis inducing factor (AIF) and Endonuclease G (Endo G) from the mitochondria to nucleus^30^,^31^. This sheds new light on overcoming the drug resistance for the tumours which are not sensitive to caspase dependent apoptosis. Therefore, inducing active cell apoptosis shows immense value in tumour therapy.

Reactive oxygen species (ROS) are oxygenic reactive chemical compounds formed as natural by-products of cellular metabolism^32^. ROS are two-edged swords in maintaining cellular homeostasis, which not only induce antimicrobial defence but also damage cell component^33^,^34^. The accumulations of intracellular ROS are the mechanism of most chemo-therapeutic and radio-therapeutic agents killing cancer cells^35^. SHK induces the generation of ROS and induces apoptosis in colon and hepatocellular cancer^14^,^36^. Among gastric cancer research, SHK time-dependently induced necrosis or apoptosis in gastric cancer cells via generation of ROS^37^.

In this study, we detected the anti-tumour effects of SHK on gastric cancer *in vitro* and *in vivo*. The results showed that SHK induces the generation of intracellular ROS, depolarizes the mitochondrial membrane potential (MMP), releases Cytochrome C from mitochondria and ultimately induces apoptosis via the caspase cascade. On the other hand, SHK induces caspase-independent apoptosis by the nuclear translocation of AIF and Endo G, which is first reported in our research. In addition, SHK enhances chemotherapeutic sensitivity of gastric cancer cells *in vitro* and *in vivo*. These results indicate that SHK is a novel inducer of apoptosis in gastric cancer and a promising candidate as a chemo-sensitizer for drug resistant gastric cancer patients.

## Results

### SHK suppresses proliferation and induces death of gastric cancer cells but does small impacts for gastric epithelial cells

**Figure 1:**
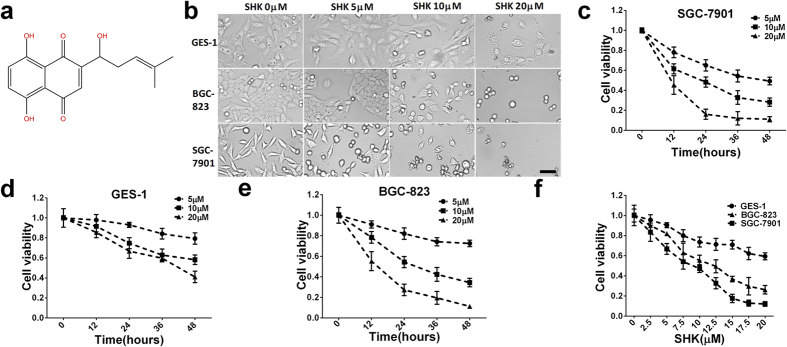
SHK inhibits cell proliferation and induces cell death. (**a**) Structure of shikonin (C_16_H_16_O_5_). (**b**) SHK induces cell death in GES-1, BGC-823 and SGC-7901 cells at 24 h as visualized by microscopy (100×, Scale bars: 50 μm). Cells were treated with increasing concentrations of SHK for 24 h (**c**) and different concentrations of SHK for different times (**d**,**e**,**f**) as indicated. Cell viability was determined by MTT assays. Values are presented as the mean ± SD of eight independent experiments for MTT and three independent experiments for flow cytometry. *P < 0.05, **P < 0.01.

The structure of SHK is presented in Fig. 1a. Immortalized gastric epithelial cell (GES-1) and gastric cancer cells (BGC-823 and SGC-7901) were treated with different concentrations of SHK. Shapes of BGC-823 and SGC-7901 cells became distorted, round and fragmented, but these effects were not significant in GES-1 cells (Fig. 1b). Cell viability detections showed that SHK suppresses cell proliferation in dose-dependent (Fig. 1c) and time-dependent (Fig. 1d,e,f) manners. The IC50 concentrations of SHK were 9.63 ± 0.43 μM (SGC-7901), 13.35 ± 0.57 μM (BGC-823) and more than 20 μM (GES-1) at 24 h. Moreover, we found that increasing concentrations of SHK caused S-phase cell cycle arrest in SGC-7901 cells (Fig. S1). Cell death was quantified by Annexin V and PI staining. SHK significantly induced death of BGC-823 and SGC-7901 gastric cancer cells, but the effects were mildly for GES-1 cells (Fig. S2). Compared with BGC-823 cells, SGC-7901 cells were more susceptible to SHK and were chosen as the main research object. In addition, 10 μM SHK was chosen as a normal experimental concentration, which was close to IC50, 5 and 20 μM SHK were regard as low and high experimental concentrations, respectively. These data indicate that SHK is a suppressor of cell growth and inducer of cell death in gastric cancer cells.

### SHK induces caspase-dependent apoptosis in SGC-7901 gastric cancer cells

**Figure 2:**
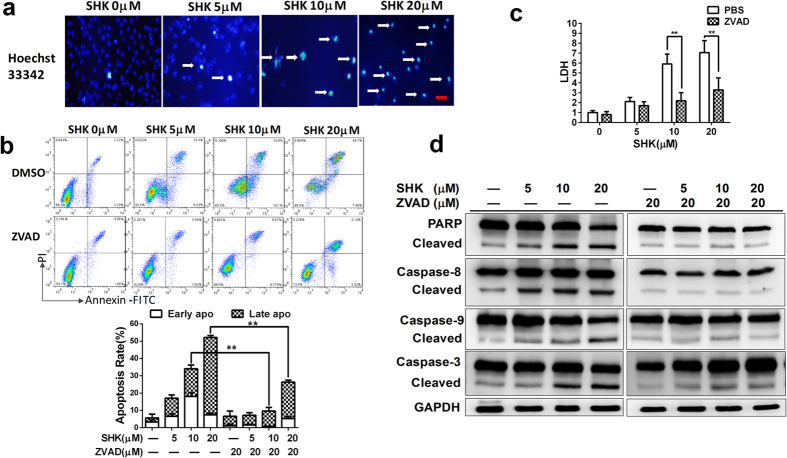
SHK-induced apoptosis is caspase-dependent in SGC-7901 gastric cancer cells. (**a**) Morphological changes of SGC-7901 cells were detected via fluorescence microscopy (100×, Scale bars: 50 μm) 24 h after SHK treatment. Arrows indicate cells with condensed nuclei. (**b**) Cell apoptosis of SGC-7901 was detected by flow cytometry 24 h after SHK treatment with or without ZVAD-FMK (20 μM). (**c**) LDH was detected 24 h after treatment with or without ZVAD-FMK (20 μM) in SGC-7901 cells. (**d**) Detection of apoptosis-related protein PARP and caspase-3, -8, -9. GAPDH was used as a loading control. Values are presented as the mean ± SD of three independent experiments. *P < 0.05, **P < 0.01.

To detect the manner of death induced by SHK, we observed chromatin condensation by Hoechst staining in SGC-7901 cells (Fig. 2a). This means that SHK induces apoptosis in SGC-7901 gastric cancer cells. Furthermore, to determine whether the apoptosis induced by SHK was caspase-dependent, the pan-caspase inhibitor ZVAD-FMK was coadministered and SHK-induced cell death was significantly inhibited (Fig. 2b). The elevated lactate dehydrogenase (LDH) induced by SHK also decreased when ZVAD-FMK was added (Fig. 2c). SHK-induced splicing events that occurred in PARP, Caspase 8, 9 and 3 were also blocked by ZVAD-FMK (Fig. 2d). These data proved that the apoptosis induced by SHK is caspase-dependent. Meanwhile, Caspase 3 inhibitor ZDEVD-FMK and Caspase 9 inhibitor ZLEHD-FMK also strongly inhibited SHK-induced apoptosis of SGC-7901 gastric cancer cells (Fig. S3). Previous report^37^ also found that SHK induced necrosis at 6 h and apoptosis at 24 h of AGS gastric cancer cells, which was time-dependent. We treated SHK for shorter time to detect whether SHK could induce necrosis in SGC-7901 gastric cancer cells. However, we did not find necrotic cells at 6 h and 12 h in SGC-7901 gastric cancer cells (Fig. S4), which may be due to the variability in cell types. Taken together, SHK activates caspase cascade and induces caspase-dependent apoptosis in gastric cancer cells.

### SHK induces caspase-independent apoptosis by nuclear translocation of AIF and Endo G

**Figure 3:**
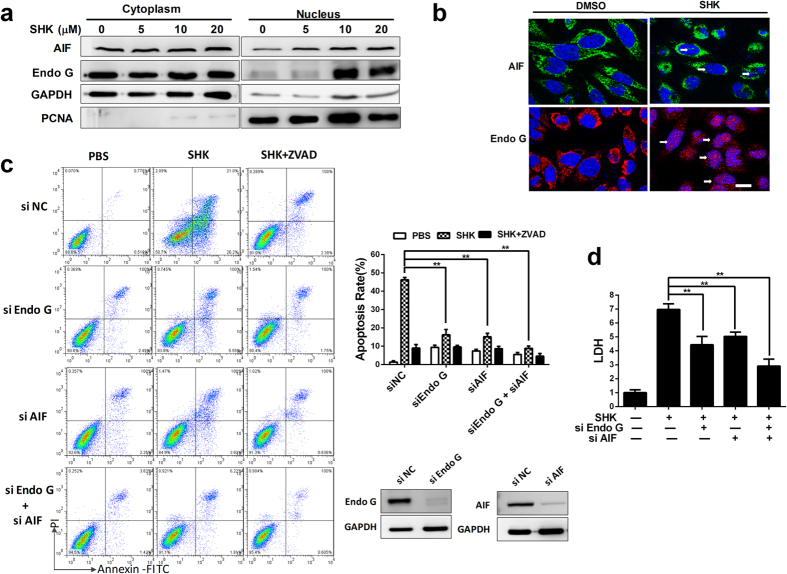
SHK induces caspase-independent apoptosis in SGC-7901 gastric cancer cells. (**a**) Western blot indicated the nuclear translocation of AIF and Endo G. GAPDH and PCNA were used as cytoplasm and nucleus loading controls respectively. (**b**) Immunofluorescence analysis of AIF (green, labelled with FITC) and Endo G (red, labelled with Cy3) localization in SGC-7901 cells 24 h after treatment of SHK. The fluorescent images were captured using a confocal microscope (600×, Scale bars: 10 μm). Cell nuclei are stained by DAPI, and arrows indicate nuclear translocation of AIF and Endo G. (**c**) The cells were transfected with siRNA for 24 h and then SHK (10 μM) was applied with or without ZVAD (20 μM). SGC-7901 cell death was analysed by flow cytometry. (**d**) LDH was detected in SGC-7901 cells 24 h after treatment with SHK (10 μM). Values are presented as the mean ± SD for at least three independent experiments. *P < 0.05, **P < 0.01.

As is reported, AIF and Endo G play critical roles in caspase-independent apoptosis^30^,^31^. These proteins translocate to the nucleus and induce DNA degradation. Western blot showed the translocation of AIF and Endo G from the cytoplasm to nucleus with treatment of SHK (Fig. 3a), and this result was reaffirmed by immuno-fluorescence staining analyses (Fig. 3b). RNA interference of AIF and Endo G was utilized (Fig. 3c). AIF and Endo G knockdown significantly inhibited SHK-induced cell death (Fig. 3c) and the elevated LDH release (Fig. 3d). Meanwhile ZVAD-FMK had synergistic effects with AIF and Endo G knockdown (Fig. 3c). We also detected the DNA fragments and found that AIF and Endo G knockdown inhibited DNA laddering (Fig. S5). These data suggested that in addition to the caspase-dependent pathway, SHK also triggers caspase-independent apoptosis that is dependent on nuclear translocation of AIF and Endo G.

### RIPK1 promotes survival of SGC-7901 cells in SHK-induced apoptosis

**Figure 4:**
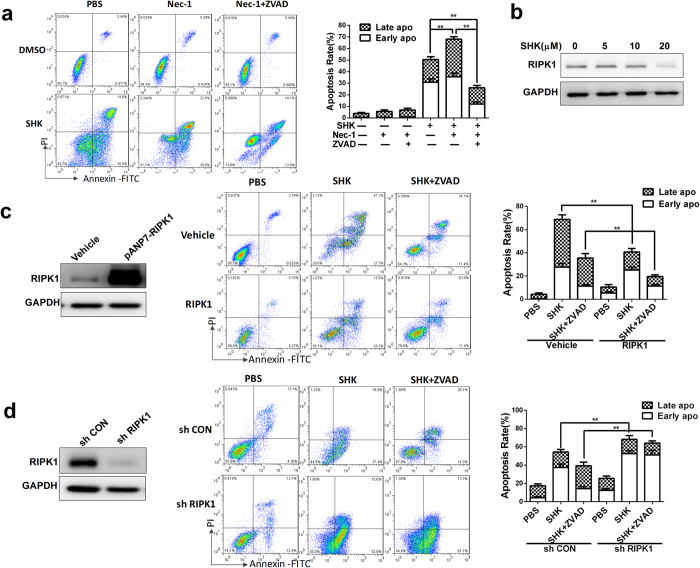
RIPK1 has a protective role in SHK-induced apoptosis. (**a**) Nec-1 (50 μM) contributes to apoptosis induced by SHK (10 μM) and these effects could be partly abolished by ZVAD-FMK (20 μM). (**b**) Western blots indicated the decrease in RIPK1 with treatment of SHK. (**c**) Cells transfected with pANP7-RIPK1 plasmids or control were incubated with SHK (10 μM) with or without ZVAD-FMK (20 μM) for 24 h. Western blots were used to determine the effects of over-expression. (**d**) Cells infected with RIPK1 shRNA lentivirus particles or control for 48 h were incubated with SHK (10 μM) with or without ZVAD-FMK (20 μM) for 24 h. Western blots were used to determine the effect of knockdown. Values are presented as the mean ± SD for at least three independent experiments. *P < 0.05, **P < 0.01.

Necroptosis is a programmed form of necrosis and SHK has been reported as a necroptosis inducer in previous studies^16^,^18^,^38^. Necroptosis is typically inhibited by the blockade of RIPK1. Although no necroptosis (PI+, Annexin V+) was found, previous reports^18^,^19^ indicated that there were necrotic cells in the late apoptosis stage of SHK-induced cell death. In addition, some reports^39^,^40^ support that inhibition of RIPK1 enhances SHK-induced apoptosis. To detect whether there are necrotic components in the late apoptosis stage or RIPK1 could regulate apoptosis in SHK-induced gastric cancer cell death, the RIPK1 inhibitor Nec-1 was used. Interestingly, Nec-1 did not inhibit but increased SHK-induced cell death (Fig. 4a). This increase of cell death was inhibited by ZVAD-FMK (Fig. 4a). These data suggest that SHK does not induce necroptosis and Nec-1 promotes SHK-induced apoptosis in SGC-7901 cells. Western blot showed that RIPK1 protein levels were decreased after treatment with SHK (Fig. 4b). We constructed over-expression plasmids and down-expression shRNA lentiviral particles of RIPK1. RIPK1 over-expression prevents SHK-induced apoptosis (Fig. 4c), whereas RIPK1 knockdown contributes to SHK-induced apoptosis (Fig. 4d). In addition, ZVAD-FMK had synergistic protective effects with RIPK1 over-expression (Fig. 4c), but RIPK1 knockdown robbed the protection of ZVAD-FMK in SHK-induced cell death (Fig. 4d). In order to detect whether RIPK1 knockdown could change cell death modality, we increased the concentration of ZVAD from 20 μM to 50 μM (Fig. S6). The high concentration of ZVAD had better protective effect, which indicated that the SHK-induced death modality in RIPK1 knockdown cells was apoptosis. Moreover, LDH levels (Fig. S7) confirmed the same results with Fig. 4c,d. In conclusion, SHK does not induce necroptosis in gastric cancer cells, and the expression of RIPK1 has a protective role in SHK-induced apoptosis.

### Intracellular ROS play an upstream role in SHK-induced apoptosis

**Figure 5:**
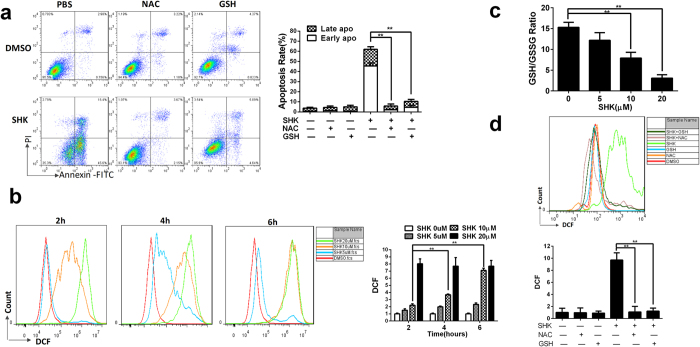
Increasing intracellular ROS levels are involved in SHK-induced apoptosis. (**a**) Apoptosis induced by SHK (10 μM) is abolished by antioxidants NAC (1 mM) and GSH (1 mM). Cell death was analysed by flow cytometry 24 h after drug treatment. (**b**) Intracellular ROS are increasing in time- and dose-dependent manners after SHK treatment. (**c**) The GSH/GSSG ratio was decreased, indicating the accumulation of ROS in a dose-dependent manner. (**d**) Increasing intracellular ROS levels induced by SHK (10 μM) were abolished by NAC (1 mM) and GSH (1 mM). All of the intracellular ROS were detected by flow cytometry using DCF probes 6 h after treatment. Values are presented as the mean ± SD for three independent experiments. *P < 0.05, **P < 0.01.

The generation of ROS exhibit a close correlation with tumour cell death^36^,^41^. To identify roles of intracellular ROS in SHK-induced gastric cancer cell death, ROS scavengers N-acetyl-L-cysteine (NAC) and L-glutathione (GSH) were used. NAC and GSH significantly inhibited SHK-induced death (Fig. 5a). ROS were measured by DCF probes and exhibited dose-dependent and time-dependent manners in response to SHK treatment (Fig. 5b). Intracellular GSH and GSSG (oxidized forms of GSH) levels reflect redox state of cells, and the GSH/GSSG ratio was significantly reduced response to SHK treatment (Fig. 5c). In addition, the antioxidants NAC and GSH completely inhibited the generation of ROS (Fig. 5d). However, ZVAD-FMK, AIF, Endo G and RIPK1 played less or no roles in the regulation of ROS (Fig. S8). These data indicate that ROS play an upstream role of caspase, AIF, Endo G and RIPK1 in SHK-induced apoptosis. ROS typically promote mitochondrial dysfunction and mitochondria-mediated apoptosis^42^,^43^,^44^. We consider that ROS play an upstream role and mediate mitochondria-related apoptosis in gastric cancer cells.

### SHK depolarizes MMP and leads to mitochondria-mediated apoptosis through ROS

**Figure 6:**
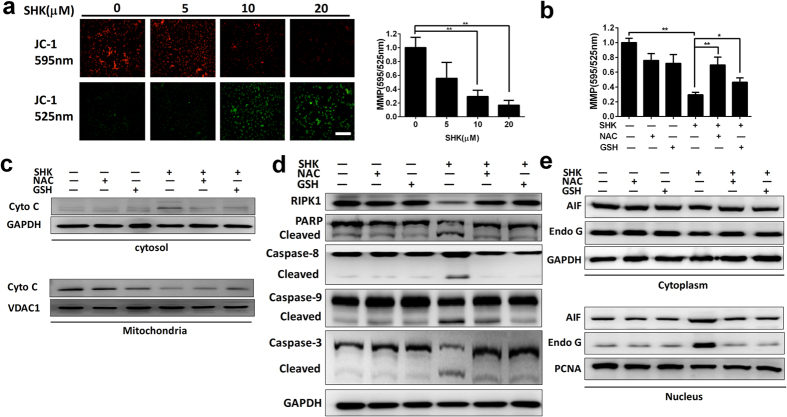
SHK induces loss of MMP, release of mitochondrial Cytochrome C, the caspase cascade and nuclear translocation of AIF and Endo G through ROS. (**a**) MMP was detected by fluorescence microscopy (40×, Scale bars: 100 μm) and flow cytometry using JC-1 probes 12 h after SHK treatment. (**b**) NAC (1 mM) and GSH (1 mM) abolished the loss of MMP 12 h after SHK treatment (10 μM). (**c**) Western blots indicated that NAC (1 mM) and GSH (1 mM) abolished the release of mitochondrial Cytochrome C. VDAC1 was used as loading control of mitochondria. (**d**) NAC (1 mM) and GSH (1 mM) abolished the decrease in RIPK1 and the cleavage of caspase-3, -8, -9 and PARP. (**e**) NAC (1 mM) and GSH (1 mM) abolished the nuclear translocation of AIF and Endo G. PCNA was used as a nucleus loading control. Values are presented as the mean ± SD for three independent experiments. *P < 0.05, **P < 0.01.

Accumulation of intracellular ROS typically depolarizes mitochondrial membrane potential (MMP)^45^. Thus, we detected the MMP of SGC-7901 gastric cancer cells after treatment of SHK. SHK depolarized MMP in a dose-dependent manner (Fig. 6a), and this effect could be blocked by ROS scavengers NAC and GSH (Fig. 6b). We also detected release of mitochondrial Cytochrome C (Fig. 6c); cleavage of caspase 8, 9, 3 and PARP (Fig. 6d); and nuclear translocation of AIF and Endonuclease G (Fig. 6e) by Western blot. However, all of these events could be partially or completely inhibited by the ROS scavengers NAC and GSH (Fig. 6c–e). The release of Cytochrome C, caspase cascade and the nuclear translocation of AIF and Endo G are the main forms of mitochondria-mediated apoptosis. These data indicate that the generation of ROS, depolarization of MMP and mitochondria-mediated apoptosis are components of a programmed event in SGC-7901 cancer cells treated with SHK.

### SHK enhances chemotherapeutic sensitivity of 5-fluorouracil and oxaliplatin

**Figure 7:**
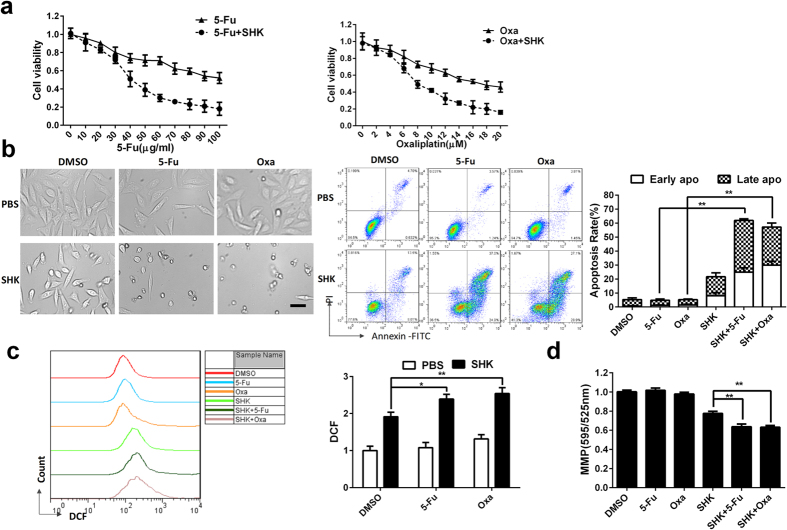
SHK enhances the chemotherapeutic sensitivity of SGC-7901 gastric cancer cells. (**a**) MTT assays were used to detect the chemotherapeutic effects of 5-Fu and Oxa alone or in combination with SHK (5 μM) 24 h after treatment. (**b**) Cell morphology was visualized by microscopy (100×, Scale bars: 50 μm), and cell death was detected by flow cytometry 24 h after treatment with 5-Fu (50 μg/ml) or Oxa (10 μM) alone or in combination with SHK (5 μM). (**c**) Intracellular ROS was detected by DCF probes at 6 h after treatment same as (**b**). (**d**) MMP was detected by flow cytometry using JC-1 12 h after treatment same as (**b**). Values are presented as the mean ± SD for three independent experiments. *P < 0.05, **P < 0.01.

5-Fluorouracil (5-Fu) and oxaliplatin (Oxa) are commonly-used chemotherapy drugs for gastric cancer. Drug resistance of cancer is a major cause of chemotherapy failure. We found that a low dose (5 μM) of SHK exhibited significantly synergistic effects with 5-Fu and Oxa (Fig. 7a). However, SHK (5 μM), 5-Fu and Oxa alone did not induce significant apoptosis, and the combination of SHK (5 μM) with 5-Fu or Oxa increased cell apoptosis dramatically (Fig. 7b). We also found that the combination of low dose SHK with 5-Fu or Oxa increases intracellular ROS (Fig. 7c) and depolarizes MMP (Fig. 7d). These data suggest that even at a low dose, SHK enhances the chemotherapeutic sensitivity of gastric chemotherapy drugs. This effect may be dependent on the accumulation of intracellular ROS and depolarization of MMP.

### SHK induces apoptosis of xenografts and enhances anticancer activities of chemotherapy drugs *in vivo*

**Figure 8:**
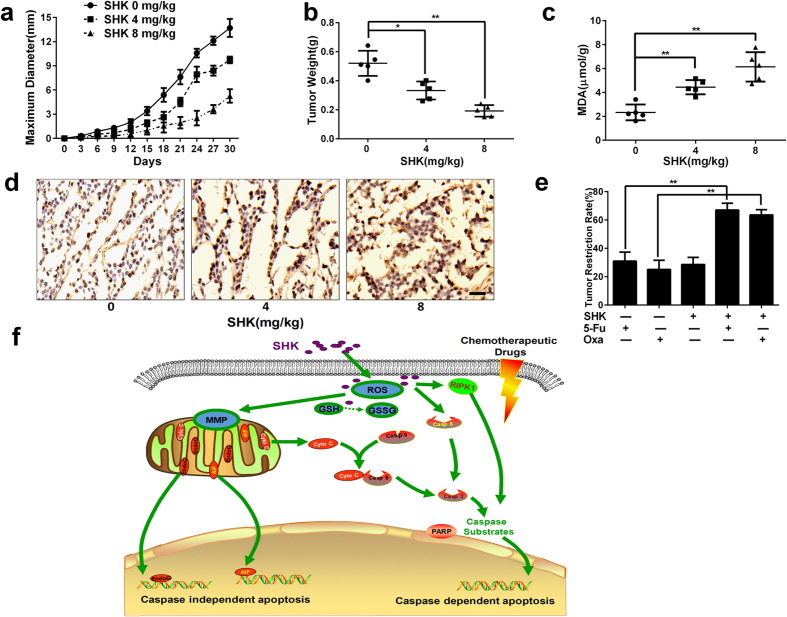
SHK inhibits SGC-7901 tumour growth and enhances chemotherapeutic sensitivity *in vivo*. (**a**) The maximum diameter of xenografts was measured by callipers every three days. The tumour-bearing models were established in BALB/c nude mice (n = 5 of each group), and then administered SHK (0, 4 and 8 mg/kg body weight) via gastric infusion. (**b**) Xenografts were weighed by microbalance 30 days after treatment with different SHK. (**c**) MDA content of xenografts was detected, which represents lipid peroxidation. (**d**) TUNEL assays were used to detect apoptosis of xenografts (40×, Scale bars: 100 μm). (**e**) Tumour restriction rate of xenograft models was calculated for the combination of 5-Fu or Oxa plus SHK. 5-Fu (20 mg/kg body weight) or Oxa (10 mg/kg body weight) was injected, and SHK (4 mg/kg body weight) was administered by gastric infusion. (**f**) Pathway of SHK-induced apoptotic cell death in gastric cancer cells as plotted by Science Slides. Values are presented as the mean ± SD for three independent experiments. *P < 0.05, **P < 0.01.

To further evaluate the anti-tumour effects of SHK in gastric cancer, we established immuno-deficient nude mice xenografts bearing SGC-7901 gastric cancer cells. The maximum diameter and weight of xenografts were measured. SHK inhibited the size and weight of xenografts (Fig. 8a and b). We detected malondialdehyde (MDA) content of xenografts, which represents lipid peroxidation, and xenografts treated with SHK exhibited ROS accumulation *in vivo* (Fig. 8c). To evaluate whether SHK could induce apoptosis in xenografts, a TUNEL assay was performed. As is shown in Fig. 8d, TUNEL-positive cells were significantly increased with increasing concentrations of SHK. In addition, the combination of SHK with 5-Fu or Oxa increased tumour restriction rates in mice (Fig. 8e), indicating that SHK also enhanced the sensitivity to chemotherapy drugs *in vivo*. Consistent with the results *in vitro*, the results suggest that SHK induces cell death and enhance anti-tumour effects of chemotherapy drugs *in vivo* experiments. Pathways of SHK-induced apoptotic cell death in gastric cancer cells are shown in Fig. 8f.

## Discussion

Cancer is the leading killer of human being with an estimated 14.1 million new cases and 8.2 million cancer deaths per year worldwide^3^. Searching for effective anti-tumour medicines has been a focus of researchers. Plants generally contain a variety of anti-tumour components, and many plant extracts have been used in the clinic to treat cancer, such as vincristine, paclitaxel, topside and topotecan. SHK is a naphthoquinone extracted from Chinese herbal medicine plant and is considered to be safe due to its long history use in Chinese traditional medicine. Recent studies confirmed its anti-tumour effects in osteosarcoma^46^, leukaemia^39^, colon cancer^14^, breast cancer^16^ and glioma^19^. In this study, we found that SHK suppresses proliferation and induces death in SGC-7901 and BGC-823 gastric cancer cells in dose- and time-dependent manners. In contrast, this agent has minimal effects on immortalized gastric epithelial GES-1 cells. Kim *et al*. demonstrated that SHK induces cell cycle arrest in human AGS gastric cancer cells^21^ and we also found that SHK caused S-phase cell cycle arrest in SGC-7901 and BGC-823 gastric cancer cells. Previous study also supported that SHK inhibits the cell viability, adhesion, invasion and migration of the human gastric cancer cell line MGC-803^47^. These data demonstrated the anti-gastric cancer effects of SHK.

ROS are double-edged swords for tumour. On the one hand, ROS cause severe DNA damages and gene mutations and active inflammation, proliferation, invasion, metastasis and angiogenesis of tumour cells^48^. On the other hand, once the levels of ROS reach the threshold, tumour growth is suppressed, cell structures break down and the cancer cells die. This is the mechanism by which most chemo-therapeutic and radio-therapeutic agents kill cancer cells^35^. In our study, we found that SHK induces the generation of intracellular ROS in time- and dose-dependent manners, depolarizes the MMP and ultimately induces mitochondria-mediated apoptosis. GSH is an important antioxidant in cellular metabolism and capable of preventing damages caused by ROS. Once oxidized, GSH is converted to its oxidized form GSSG. The ratio of GSH/GSSG is a measure of cellular oxidative stress^49^. We found that the GSH/GSSG ratio was significantly decreased when treated with SHK. In addition, as ROS scavengers, GSH and NAC inhibited SHK-induced cell death. However, the pan-caspase inhibitor ZVAD-FMK and knockdown of AIF and Endo G do not affect intracellular ROS, indicating that ROS play an upstream role in SHK-induced apoptosis.

The accumulation of ROS and depolarization of MMP may cause mitochondrial swelling and increase permeability of the mitochondrial membrane, which induces intrinsic apoptosis (also called mitochondria-mediated apoptosis)^28^. On the one hand, Cytochrome C leaks out from the perforated mitochondria and binds to apoptotic protease activating factor-1, forming apoptosomes. Then, apoptosomes activate caspase 9 and initiate the chain reaction of caspase cascade. These processes are defined as caspase dependent-apoptosis^50^. On the other hand, AIF and Endo G are released from perforated mitochondria, translocated into the nucleus and induce chromosomal DNA fragmentations. These processes are defined as caspase-independent apoptosis^51^. Our data showed that SHK induces apoptosis of gastric cancer cells via both the caspase-dependent and -independent pathways. SHK induces apoptotic cell morphology, release of Cytochrome C from mitochondria and cleavage of caspase 3, -9 and PARP, which is blocked by ZVAD-FMK. In addition, AIF and Endo G are translocated from the cytoplasm to the nucleus, which was affirmed by Western blots and immunofluorescence assays, and knockdown of AIF and Endo G inhibit SHK-induced apoptosis. These data confirm that SHK induces caspase-independent apoptosis in gastric cancer cells. Many cancer cells are not sensitive to drugs due to caspase escape and caspase-independent apoptosis is a new strategy to overcome drug resistance. In addition, we also observed the activation of caspase 8, indicating that extrinsic apoptosis (death receptor signaling pathway) may be involved in SHK induced-apoptosis. This finding must to be further confirmed.

RIPK1 is an enzyme that plays an important role in the NF-κB pathway, apoptosis and necroptosis. In TNFR1-elicited signal pathways, RIPK1 is a constituent component of complex I, which includes RIPK1, TRADD, cIAPs, TRAF2 and TRAF5. In cIAP-mediated ubiquitylation, RIPK1 initiates NF-κB pathways, which promote cell survival. RIPK1, RIPK3, caspase 8, TRADD and FADD form complex II. Caspase 8 cleaves RIPK1 and RIPK3 and triggers the caspase cascade to induce apoptosis. When caspase 8 is blocked, RIPK1 and RIPK3 become phosphorylated and trigger necroptosis^52^. Recent studies reported that RIPK1 mediates necroptosis induced by SHK in osteosarcoma, breast cancer and glioma^16^,^18^,^19^. According to previous report of Lee *et al*., SHK induced necrosis at 6 h and apoptosis at 24 h of gastric cancer cell AGS, which was time-dependent^37^. In our study, we did not find necrotic cells at 6 h and 12 h in SGC-7901 gastric cancer cells. Interestingly, we demonstrated that RIPK1 inhibitor Nec-1 does not rescue but increases SHK-induced cell death. RIPK1 Over-expression prevents SHK-induced cell death in gastric cancer cells, whereas RIPK1 knockdown contributes to it. These data suggest that SHK may not trigger RIPK1-dependent necroptosis in gastric cancer cells and RIPK1 plays a prosurvival role in SHK-induced apoptosis.

The ultimate aim of developing novel anti-tumour drugs is clinical application and developing chemo-sensitive strategies and agents is of great importance^8^. Multi-drug combinations are a novel approach to overcome drug resistance. We evaluated the effect of SHK combined with 5-Fu or Oxa and found that low dose SHK enhances anti-tumour effects. The molecular mechanisms of this synergistic effect may be involve the accumulation of ROS and depolarization of MMP. Furthermore, data also suggest that SHK suppresses tumour growth in xenograft animal models. SHK increases lipid peroxidation and induces apoptosis *in vivo*, which is consistent with the results *in vitro*. In addition, SHK enhances the anti-tumour activities of 5-Fu and Oxa *in vivo*.

In conclusion, SHK is an intracellular ROS inducer and we demonstrated its ability to suppress cell proliferation. SHK induces apoptosis in gastric cancer cells via caspase-dependent and -independent manners, which has never been reported in previous studies. In addition, we also demonstrate that SHK enhances chemotherapeutic sensitivity in gastric cancer both *in vitro* and *in vivo*. Therefore, this study suggests that SHK may be a novel therapeutic agent in the clinical treatment of gastric cancer.

## Methods

### Cell culture

Human gastric cancer cell lines BGC-823, SGC-7901 and normal human gastric mucosal epithelial cell line GES-1 were purchased from the Institute of Basic Medical Sciences Chinese Academy of Medical Sciences. Cells were cultured in RPMI 1640 medium (Gibco, NY, USA) with 10% fetal bovine serum (HyClone, UT, USA) in a cell incubator with an atmosphere of 5% CO_2_ at 37 °C.

### Chemicals and antibodies

SHK was purchased from Sigma-Aldrich (St. Louis, MO, USA), dissolved in DMSO (Sigma-Aldrich, St. Louis, MO, USA). ZVAD-FMK, Necrostatin-1, 5-Fluorouracil and Oxaliplatin were from MedChemExpress (NJ, USA). ZDEVD-FMK and ZLEHD-FMK were from Adooq bioscience (CA, USA). N-acetyl-L-cysteine and L-glutathione were from Beyotime Institute (Shanghai, China). Antibodies were as follows: PARP, Caspase 3, Caspase 8, Caspase 9, RIPK1, Cytochrome C and VDAC1 were from Cell Signaling Technology (MA, USA); AIF, Endonuclease G, GAPDH and PCNA were from Abcam (Cambridge, UK); the goat IgG-HRP secondary antibodies against rabbit and mouse were from Zhongshan Golden Bridge Biotechnology (Peking, China) and FITC or Cy3 conjugated secondary antibodies were from Sigma (MO, USA).

### Determination of cell viability and cell death

MTT assay (Roche, Mannheim, Germany) was used to detect cell viability following the manufacturer’s instructions. Cells were seeded in 96-well plates, cultured overnight and treated with drugs. MTT (5 mg/ml, Sigma) was added for 4 hour-incubation, removed supernatant and solubilized MTT using DMSO (Sigma, USA). The absorbance was measured by a 96-well plate reader (Bio-Tec Instrument) at 490 nm. Hoechst 33342 staining (Sigma-Aldrich, St. Louis, MO, USA) was used to analyze the nuclear morphology. Cells were incubated with Hoechst 33342 (5 μg/ml, Sigma) and PI (10 μg/ml, Sigma) at 37 °C, then washed in PBS and visualized with a fluorescence microscope (Nikon, Japan). Annexin V and PI staining kit (Beyotime Biotechnologies, Jiangsu, China) was applied to quantify the cell death. After treatment, cells were washed with PBS, resuspended in 200 μl binding buffer, incubated with 5 μl Annexin V-FITC and 10 μl PI for 20 min at room temperature in the dark. The stained cells were quantified by flow cytometer (BD bioscience, USA).

### Cell cycle analysis

Cells were seeded in 6-well plates and treated with shikonin for 24 h, then collected, washed with cold PBS and fixed with 70% ethanol overnight at 4 °C. Cells were incubated with PI (50 μg/ml, Sigma) and RNase (100 μg/ml, Sigma) for 1 h, then analysed by flow cytometer (BD bioscience, USA).

### LDH assay

LDH assay (Beyotime Biotechnologies, Jiangsu, China) was used to detect the cytotoxicity according to the manufacturer’s protocol. Briefly, after treatment the media supernatants were transferred to a 96-well plate, added 50 μl LDH detection reagent, incubated for 1 hour at 37 °C, added chromogenic agent, finally the absorbance was measured by a 96-well plate reader (Bio-Tec Instrument).

### Isolation of nuclei and mitochondria

A nuclear/cytosol fractionation kit (Beyotime Biotechnologies, Jiangsu, China) was used to isolate the nuclei according to the manufacturer’s protocol. Briefly, cells were collected, resuspended with cytosol extraction reagent by vigorous vortex mixing for 30 min and centrifuged. The supernatant was collected as cytosolic fraction and the pellet was collected as nuclear fraction.

A mitochondria isolation kit (Beyotime Biotechnologies, Jiangsu, China) was used to isolate the mitochondria according to the manufacturer’s protocol. Briefly, cells were collected, resuspended with extraction buffer, incubate on ice, homogenized the cells using a homogenizer for 30 strokes and centrifuged the homogenate at 600 g for 10 min at 4 °C. The supernatant was carefully transferred to a fresh tube and centrifuged at 11,000 g for 10 min at 4 °C. The supernatant was collected as cytosolic fraction and the pellet was collected as mitochondria fraction.

### Immunofluorescence staining analyses

After treatment, cells were fixed with 4% paraformaldehyde, permeabilized with 0.5% triton X-100 for 15 min, blocked with PBS buffer containing 5% bovine serum albumin and washed with PBS three times. The cells were incubated with primary antibodies overnight at 4 °C and followed by 2 hour-incubation with FITC or Cy3 conjugated secondary antibodies. The nuclear was stained with DAPI (Sigma, USA) and images of immunostained cells were captured with a confocal microscope (Nikon, Japan) using the ×60/1.35 NA oil objective.

### RNA interference, plasmid transfection and lentivirus infection

The siRNA against Endo G (target sequence of 5′-CCAUGGACGACACGUUCUA-3′), AIF (target sequence of 5′-GCAGUGGCAAGUUACUUAU-3′) and control siRNA (target sequence of 5′-UUCUCCGAACGUGUCACGU-3′) were synthesized by GenePharma Technologies (Shanghai, China). The RIPK1 plasmids were synthesized by RT-PCR and identified by gene sequencing. The RIPK1 shRNA sequence (GCAAAGACCTTACGAGAATTT) was synthesized by Genwiz Technologies (Suzhou, China) and constructed with pLKO.1 lentiviral vector plasmids. Procedures of RNA interference, plasmid transfection and lentivirus infection are previously described^53^.

### DNA laddering

DNA ladder was extracted from cells after treatment with Qiagen DNA Extraction Kit (Germany) by the manufacturer’s recommendations and then visualized in an agarose gel by ethidium bromide staining.

### Measurement of intracellular ROS level and mitochondrial membrane potential

A Reactive Oxygen Species Assay Kit (Beyotime Technologies, Jiangsu, China) was used by the manufacturer’s recommendations. After treatment, the cells were collected and incubated with DCFH-DA at 37 °C in the dark. DCF fluorescence intensity was measured by flow cytometry (BD bioscience, USA). And a Mitochondrial Membrane Potential Assay Kit (Beyotime Technologies, Jiangsu, China) according to the manufacturer’s recommendations. After treatment, cells were incubated with JC-1 for 30 min at 37 °C in the dark. Then cells were visualized with a fluorescence microscope (Nikon, Japan) or quantified by FACS Calibur flow cytometer (BD bioscience, USA).

### Measurement of intracellular GSH/GSSG

A GSH and GSSG Assay Kit (Beyotime Technologies, Jiangsu, China) was used according to the manufacturer’s recommendations. After treatment, cell lysates were obtained by three freeze-thaw cycles in liquid nitrogen. The mixture was centrifuged and the supernatant was collected for GSH and GSSG measurement.

### Western blot

Whole cells and subcellular components were suspended in Laemmli Buffer (Bio-Rad Laboratory, USA). Protein concentrations were measured using the BCA Protein Assay Kit (Pierce, USA). Equal amounts of protein were separated by SDS-PAGE and transferred to PVDF membrane (Bio-Rad Laboratory, USA). The membrane was blocked with 5% skim milk, and incubated with primary antibodies overnight at 4 °C and secondary antibodies for 2 hours at room temperature.

### Establishment of xenografts

Five-week-old BALB/c nude mice were purchased from Vital River (Beijing, China). Procedures involving animals and their care were conducted in conformity with NIH guidelines and was approved by Animal Care and Use Committee of Chinese PLA General Hospital. SGC-7901 cells were harvested, and a mixture of 5 × 106 cells, PBS and matrigel (Corning, NY, USA) was injected subcutaneously in the right oxter. After the xenografts were established, the tumour-bearing models were administered SHK via gastric infusion combined with or without 5-Fu or Ox via intraperitoneal injection. The diameter of xenografts was measured by calipers every three days. One month later, the mice were sacrificed, and tumours were weighed. The tumour restriction rate was calculated as follows: (1-Tumour Weight of Test Sample/Tumour Weight of Control) × 100%.

### MDA and TUNEL assays

MDA Assay Kit (Beyotime Technologies, Jiangsu, China) was used to detect the malondialdehyde (MDA) content of xenografts according to the manufacturer’s protocols. Tumour were homogenized by sonication extraction and normalized by BCA Protein Assay Kit (Pierce, IL, USA). A TUNEL Assay Kit (Roche, IN, USA) was used to detect the apoptosis of xenografts according to the manufacturer’s protocols. Tumours were fixed by formalin and embedded by paraffin. DNA strand breaks were identified by TUNEL probes.

### Statistical analysis

Values are presented as the mean ± SD for at least three independent experiments. Statistical comparisons were analysed by using one-way ANOVA with GraphPad Prism 6 (La Jolla, CA, USA). P < 0.05 indicates a significant difference and P < 0.01 indicates an extremely significant difference.

## Additional Information

**How to cite this article**: Liang, W. *et al*. Shikonin induces mitochondria-mediated apoptosis and enhances chemotherapeutic sensitivity of gastric cancer through reactive oxygen species. *Sci. Rep.* **6**, 38267; doi: 10.1038/srep38267 (2016).

**Publisher’s note:** Springer Nature remains neutral with regard to jurisdictional claims in published maps and institutional affiliations.

## Supplementary Material

Supplementary Figures
