# Supplementary material for: Shikonin induces mitochondria-mediated apoptosis and enhances chemotherapeutic sensitivity of gastric cancer through reactive oxygen species
Source: Sci Rep. 2016 Dec 1;6:38267. doi: 10.1038/srep38267 (PMC5131274; doi:10.1038/srep38267)

Supplementary Figures

Shikonin induces mitochondria-mediated apoptosis and enhances chemotherapeutic sensitivity of gastric cancer through reactive oxygen species

Wenquan Liang[1,†], Aizhen Cai[1,†], Guozhu Chen[2,†], Hongqing Xi[1,†], Xiaosong Wu[1], Jianxin Cui[1], Kecheng Zhang[1], Xudong Zhao[1], Jiyun Yu[2,*], Bo Wei[1], Lin Chen[1,*]

[1]Department of General Surgery, Chinese People's Liberation Army General Hospital, Beijing 100853, P. R. China. [2]Department of Frontier for Biological Treatment, Beijing Institute of Basic Medical Science, Beijing, 100850, China. [†] These authors contributed equally to this work. Correspondence and requests for materials should be addressed to L.C. (E-mail: linchenbj@163.com) or J.Y. (E-mail: yujyunbj@163.com).

Supplementary Figure S1

SHK induced accumulation of cells in S-phase after 24 h. Cell cycle of SGC-7901 was analysed by flow cytometry.

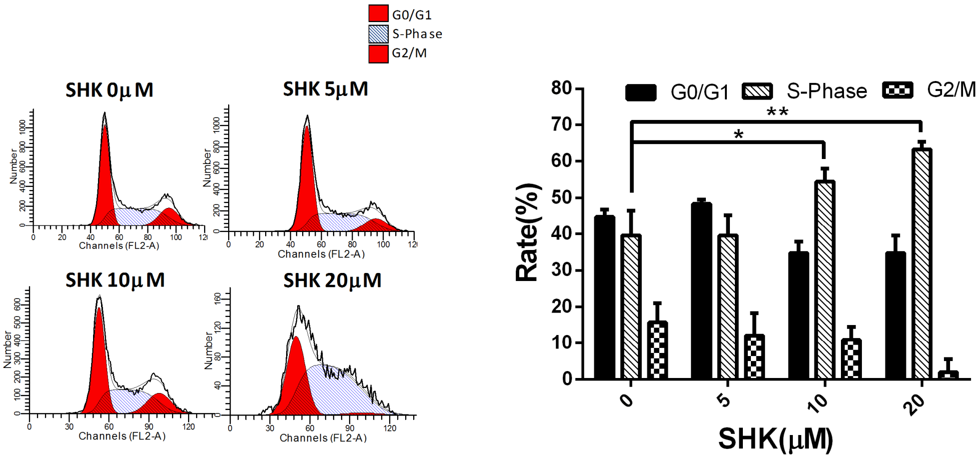

Supplementary Figure S2

Cell death of GES-1, BGC-823 and SGC-7901 induced by SHK was analysed by flow cytometry 24 h after treatment.

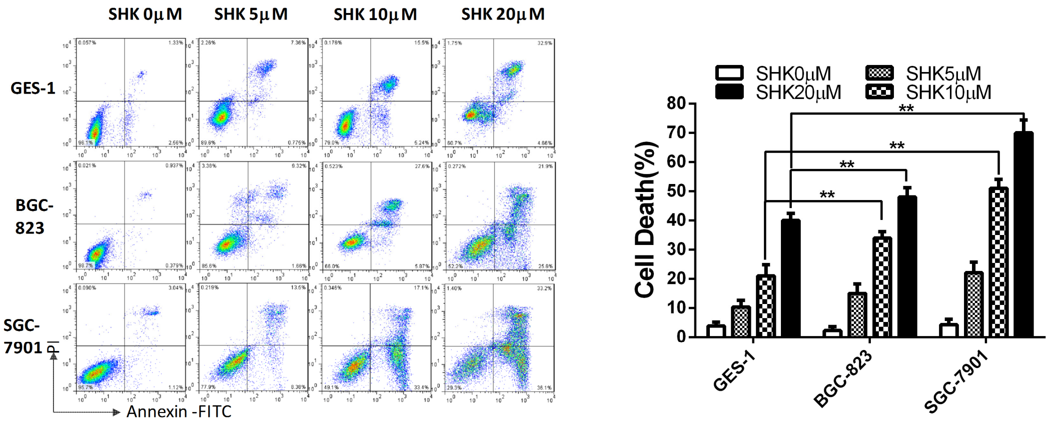

Supplementary Figure S3

The caspase 3 inhibitor ZDEVD-FMK (20 μM) and caspase 9 inhibitor ZLEHD-FMK (20 μM) were used to detect their protection of SHK (10 μM)-induced apoptosis. Data were analysed by flow cytometry 24 h after treatment.

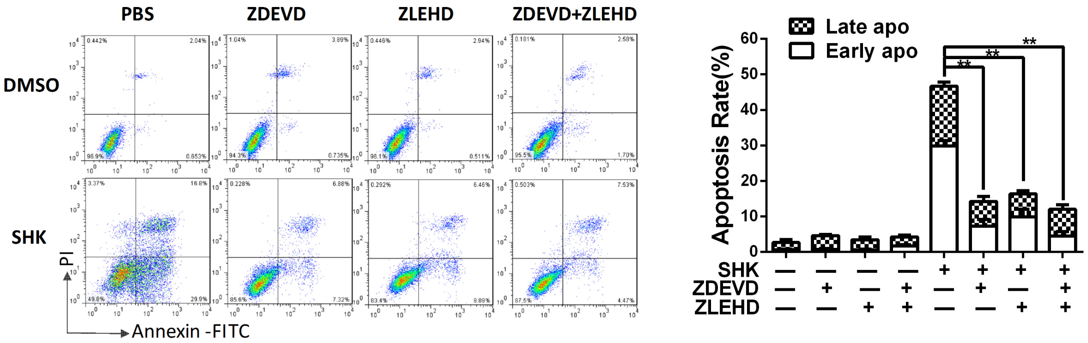

Supplementary Figure S4

Cell death of SGC-7901 gastric cancer cells was analysed by flow cytometry at 6h and 12h after SHK treatment with different concentration.

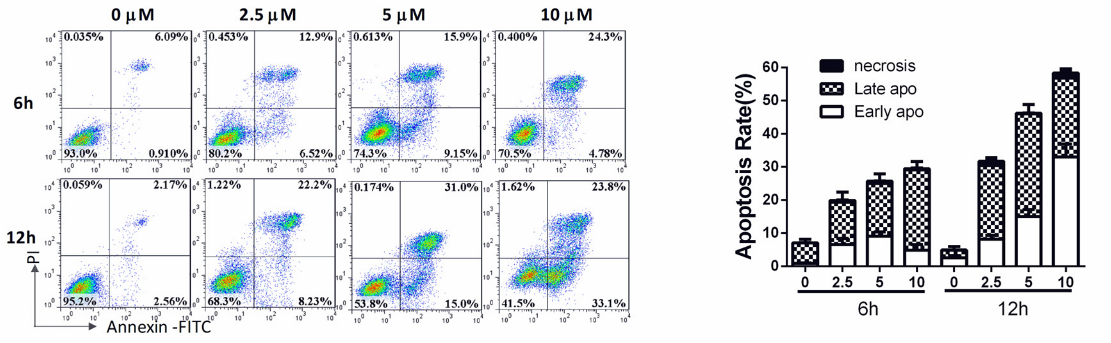

Supplementary Figure S5

DNA laddering in SGC-7901 cells showed that AIF and Endo G knockdown reduced DNA characteristic ladder fragmentation after SHK (10 μM) treatment for 24h.

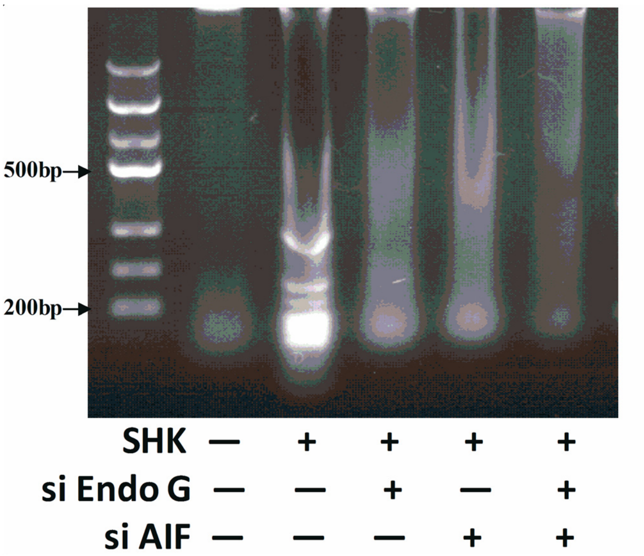

Supplementary Figure S6

Cell death of SGC-7901 gastric cancer cells with RIPK1 knockdown was analysed by flow cytometry after SHK (10 μM) with or without the co-treatment of ZVAD (50 μM) for 24h.

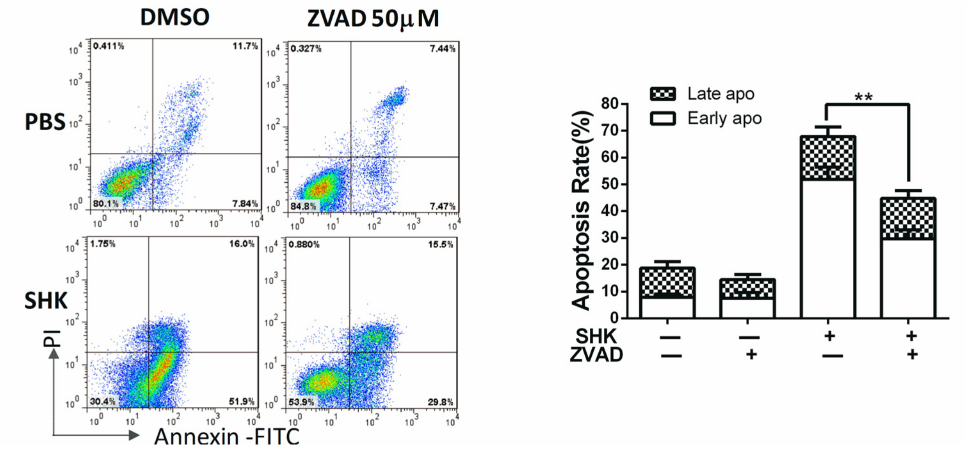

Supplementary Figure S7

LDH was detected 24 h after treatment with SHK (10 μM) to analyze the effects of RIPK1 overexpression or knockdown.

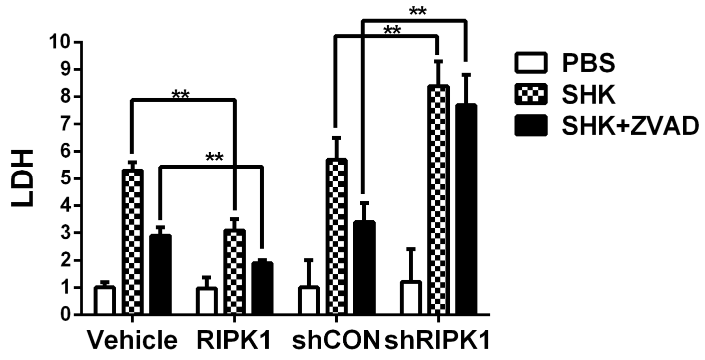

Supplementary Figure S8

ZVAD-FMK (20 μM), AIF and Endo G, and RIPK1 did not change the levels of intracellular ROS induced by SHK (10 μM). All of the intracellular ROS was detected by flow cytometry using DCF probes 6 h after treatment.

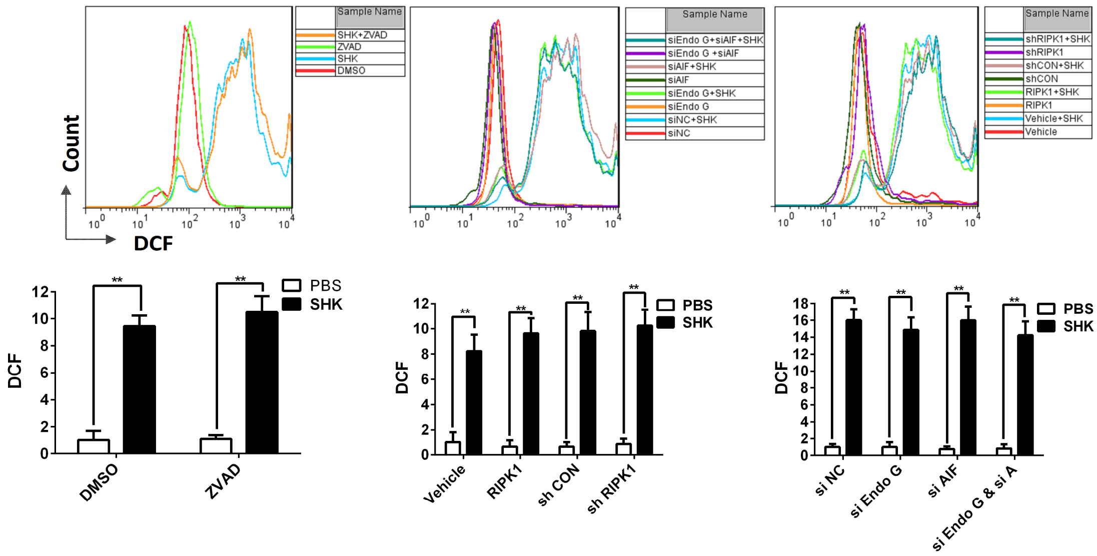

Supplement: Supplementary Figures [file srep38267-s1.pdf]
